# The spread of *Kalmia angustifolia* on black spruce forest cutovers contributes to the spatial heterogeneity of soil resources

**DOI:** 10.1371/journal.pone.0198860

**Published:** 2018-06-21

**Authors:** Gilles D. Joanisse, Robert L. Bradley, Caroline M. Preston

**Affiliations:** 1 Centre d’enseignement et de recherche en foresterie de Sainte-Foy Inc. (CERFO), Québec city, Québec, Canada; 2 Département de Biologie, Université de Sherbrooke, Boulevard de l’université, Sherbrooke, Québec, Canada; 3 Canadian Forest Service, Pacific Forestry Centre, Victoria, British Columbia, Canada; Technical University in Zvolen, SLOVAKIA

## Abstract

*Kalmia angustifolia* is a boreal ericaceous shrub that can rapidly spread on black spruce forest cutovers in eastern Canada, where CPRS (i.e. Cutting with Protection of Regeneration and Soils”) is practiced. The proliferation of *Kalmia* often coincides with a reduction in the growth rate of regenerating black spruce seedlings. We report on a study where we compared the local effects of *Kalmia* and black spruce seedling patches (i.e. two types of “Vegetation”) on chemical and biochemical soil properties in CPRS cutovers within mesic spruce-moss and xeric spruce-lichen ecosystems, as well as in four mature spruce-moss forests (i.e. three “Site Types”). Results from ^13^C-CPMAS-NMR revealed lower O-alkyl C (i.e. carbohydrates), higher aromatic C (i.e. lignin and other phenolics) and higher carbonyl-C (i.e. amide-C and carboxyl groups) in spruce-moss than in spruce-lichen forest floors (F-horizon). In spite of these distinctions, we observed only a small number of Site Type x Vegetation interactions controlling soil properties. Vegetation had a significant effect on ten forest floor properties. Most notably, *Kalmia* patches had higher concentrations of condensed tannins and lower mineral N cycling. On the other hand, Site Type had a relatively greater effect on the deeper podzolic-B horizons, where mineral N and microbial activity were higher in mature spruce-moss forests than in the cutovers. Green and senescent *Kalmia* leaves collected at these sites had higher N, tannin and phenolic concentrations than green and senescent spruce needles. A 25 month litter bag study found lower decomposition of *Kalmia* leaf litter in spruce patches on spruce-lichen cutovers compared to spruce patches on spruce-moss cutovers, or to *Kalmia* patches on spruce-lichen cutovers. Given that black spruce seedlings obtain most of their nutrients from the forest floor, our results suggest that CPRS may have long-term negative effects on black spruce forest productivity if the spread of *Kalmia* is left unchecked.

## Introduction

Black spruce (*Picea mariana* (B.S.P.) Mill.) is the most common boreal tree species and a major resource for the pulp and paper industry in Quebec, Canada. This slow-growing shade-tolerant species dominates two common ecosystem types. The spruce-moss type consists of closed-canopy black spruce stands that develop on moist soils (i.e. mesic) with a feather moss (*Pleurozium schreberi* (Brid.) Mitt) mat and an organic forest floor typically >10 cm thick. By contrast, the spruce-lichen type consists of open-canopy black spruce woodlands that develop on well-drained soils (i.e. xeric) with a ground cover of fruticose lichens (e.g. *Cladina* spp. and *Cladonia* spp.) and organic forest floors that are typically 5–7 cm thick. In both of these ecosystem types, CPRS (i.e. Cut with Protection of Regeneration and Soils) has been the principal method of timber extraction for the past 30 years. CPRS (i.e. “careful logging”) removes merchantable black spruce stems while leaving the understory (including advance regeneration of black spruce) and soils intact over 75% of the cutover area [1]. Some have argued in favor of CPRS [2], while others have criticized this practice in its intended purpose of maintaining ecological integrity and site productivity, namely by promoting the proliferation of ericaceous shrubs [3]. It is thus important to understand and compare soil properties and nutrient cycling in spruce-moss and spruce-lichen CPRS cutovers.

It is probable that some of the understory species on CPRS cutovers might decline, whereas others might benefit, from post-disturbance conditions. This is the case for ericaceous shrubs such as *Kalmia angustifolia* (L.), *Rhododendron groenlandicum* (Oeder–Kron & Judd) and *Vaccinii* spp., which tend to expand following CPRS. These shrubs first establish themselves in the understory of maturing conifer stands and come to dominate the understory in the latter stages of forest succession, as canopy gaps appear. Removal of light limitation by CPRS permits the rapid proliferation of new stems, which grow as clumps arising from rhizomatous buds. The vegetative spread of these shrubs may also be enhanced by the presence of lignin-rich forest floors and woody debris [4], both of which are prominent on CPRS cutovers. However, a recent demographic study suggests that the expansion of these shrubs might be slower in rapidly drained soils [5], such as in spruce-lichen ecosystems.

The expansion of *Kalmia* on CPRS cutovers may also coincide with reduced resource usage by black spruce seedlings. What results is referred to as conifer “growth check,” a condition that may foreshadow a drastic reduction in forest cover, retrogressive succession and subsequent heath development [6–8]. Several mechanisms have been proposed to explain *Kalmia*-induced growth check. For example, *Kalmia* may compete directly with black spruce for water and nutrients [9,10] or interfere indirectly with black spruce growth through allelopathy [11]. Perhaps the most compelling explanation for *Kalmia*’s competitive ability involves the production of condensed tannins and other phenolic compounds, which can reach up to 25% of leaf dry mass on CPRS cutovers [7]. *Kalmia* tannins released as leaf litter may form stable precipitates with soil proteins and thereby reduce the cycling of important plant nutrients such as N [12]. *Kalmia* tannins may also inhibit the activity of extracellular soil enzymes, further reducing decomposition and the availability of soil nutrients. It remains unclear, however, whether these changes in nutrient availability and soil enzyme activity has an impact on microbial activity and biomass, as different studies on this matter have shown contradictory results [6,13,14]. We posit that *Kalmia* clumps that develop on CPRS cutovers will form humus patches of lower fertility and different biochemical quality than humus found elsewhere on these cutovers [15,16].

The conversion of productive conifer stands to heathlands following disturbance may depend on the extent to which nutrient cycling is regulated by the ericaceous shrub community relative to the regenerating conifers [17]. If, for example, site fertility is high enough to allow conifer canopy closure, the resulting shade in the understory should reduce the vigour and growth of ericaceous shrubs as well as the production of tannins [4,17]. This should lead to nutrient cycling rates that sustain the growth of the coniferous stand. If, on the other hand, ericaceous shrubs come to dominate a cutover site, this could trigger a positive feedback loop involving higher soil tannin concentrations, soil enzyme inhibition, lower soil N mineralisation rates and lower leaf litter decomposition rates [7,10]. This in turn should increase the competitive ability of ericaceous shrubs, given their ability to form associations with ericoid mycorrhizae that can absorb sequestered nutrients that are unavailable to conifers [16]. Currently, there are no data to suggest the extent to which these mechanisms occur in spruce-moss vs. spruce-lichen cutovers.

We report on a study where we describe differences in forest floor C chemistry of spruce-moss and spruce-lichen cutovers, based on ^13^C nuclear magnetic resonance spectroscopy. We then compared a suite of chemical, biochemical and microbial soil properties under patches of *Kalmia* and patches of black spruce seedlings occurring on cutovers in each ecosystem type. The same was done in mature and productive spruce-moss forest stands, which stood as a reference ecosystem. We tested whether vegetation patches and site type could be predicted using aggregate properties of forest floors and mineral soil horizons. We then tested the effect of vegetation and site type on individual soil properties, on *Kalmia* tissue chemistry and on *Kalmia* leaf decomposition rates. More specifically, we hypothesized that condensed tannins would be higher and nutrient cycling lower in *Kalmia* than in black spruce patches, and that this phenomenon would be more prevalent in spruce-moss than in spruce-lichen ecosystems. We further hypothesized that the effect of vegetation would be stronger than the effect of site type in the forest floor, with possibly the opposite occurring in the underlying mineral soil horizons. The ecological implications of our results are discussed in terms of their relevance to forest management.

## Materials and methods

### Field sites

Our study was conducted in the southwestern boreal region of Québec, Canada, within a 75 km radius around the town of Senneterre (48°23.5′N, 77°14.3′W). Although the area falls within the balsam fir–paper birch bioclimatic region [18], the landscape incorporates both spruce-moss and spruce-lichen ecosystems. Soils of the region are mainly classified as Humo-Ferric Podzols according to the Canadian Soil Classification System [19], which is akin to the Umbric Podzol soil group of the International Soil Classification System [20]. Mean annual temperature and precipitation in the study area are respectively 0.5°C and 972 mm [21].

A single plot (50 m x 50 m) was established on each of 16 clearcut sites, more specifically on eight mesic spruce-moss sites and on eight xeric spruce-lichen sites. All sites have developed on undifferentiated fluvio-glacial till and differences in soil moisture arise mainly from topography. All sites had been harvested 10 years earlier by CPRS. Similar plots were established in four additional mature spruce-moss stands. These four stands were used as a reference benchmark to compare with the cutover sites, as they are assumed to reflect nutrient cycling characteristics of a black spruce-dominated overstory. Time constraints prevented us from sampling mature spruce-lichen stands and achieving a balanced sampling design. The four mature spruce-moss stands had an average canopy openness varying between 12–20%, according to 25 hemispherical canopy photos taken at each site. Ericaceous understory shrubs across sites consisted of *Kalmia*, Labrador tea, velvetleaf blueberry (*Vaccinium myrtilloides* Michaux), lowbush blueberry (*V*. *angustifolium* Aiton) and American wintergreen (*Gaultheria procumbens* L.). *Kalmia* dominated the ericaceous shrub layer in all 20 sites.

### Chemical structure of forest floor carbon

Prior to our intensive soil and vegetation sampling campaign (described below), we explored the general C chemistry of forest floor material in each of the three site types using ^13^C nuclear magnetic resonance spectroscopy with cross-polarization and magic-angle spinning (^13^C CPMAS NMR). Given time and cost constraints, it was only possible to analyze a single bulked sample from each of three spruce-moss and three spruce-lichen cutovers, and from only one mature spruce-moss stand. At each of these seven sites, blocks (625 cm^2^ x 5–10 cm thick) of forest floor (F layer) material were collected every 5 m along two randomly established 50 m transects. These forest floor samples were pooled within each site and sieved to pass a 5 mm mesh before they were analyzed.

Solid-state ^13^C NMR spectra of the seven forest floor samples were generated using a Bruker MSL 300 spectrometer (Bruker Instruments Inc., Karlsruhe, Germany) operating at 75.47 MHz. Freeze-dried and finely-ground subsamples were spun at 4.7 kHz in 7 mm dia. zirconium oxide rotors. Spectra were acquired with a 1 ms contact time, 2 s recycle time and 8000 scans. Spectra were processed using a 30 Hz line-broadening and baseline correction in Win-NMR 6.0 (Bruker Instrument Inc., Germany). Chemical shifts are reported relative to tetramethylsilane (TMS) at 0 ppm, with the reference frequency set with adamantane.

The spectral divisions were based on previously published work [22,23] and were assigned on the basis of local minima in the spectra. The NMR spectra were divided into the following chemical shifts: 0 to 50 ppm attributed to alkyl C; 50 to 93 ppm attributed to methoxyl, N-alkyl (amino acid) and O-alkyl C; 93 to 112 ppm attributed to di-O-alkyl C; 112 to 140 ppm attributed to aromatic C-C and C-H; 140 to 160 ppm attributed to aromatic C-O and C-N; 160 to 187 ppm attributed to carbonyl C (carboxyl, amide and ester C). The alkyl-C to O-alkyl-C ratio (0-50/50-112) was used as an index of the decomposition stage of the forest floor material [22]. Areas of specific shift regions were determined after integration and were expressed as the percent of the total area (i.e. relative intensity). Areas were not corrected for spinning sidebands, as these were relatively small and their effects would be similar among most samples.

### Soil and vegetation sampling

On each site, forest floor F-layer material, as well as mineral soil from the Ae (entire horizon) and podzolic B (top 20 cm) horizons, were collected beneath six patches of *Kalmia* and six patches of black spruce seedlings. Patches were at least 5 m apart, with spruce patches being *Kalmia*-free and vice versa. Within each site, the six samples of each soil horizon were composited by vegetation type (i.e. *Kalmia* or black spruce) and sieved (5-mm mesh) to remove coarse debris.

At the end of the growing season (October), bulked samples (ca. 1 kg) of both green and senescent *Kalmia* leaves were randomly collected on all 20 sites. On the 16 cutover sites, five *Kalmia* plants were excavated for fine-root sampling. Bulked samples of green and senescent black spruce needles were also randomly collected on the 16 cutover sites.

The 120 bulked soil samples, the 72 bulked needle and foliar samples, as well as the 80 uprooted *Kalmia* plants were transported on ice to the laboratory and stored at 4°C prior to analyses.

### Soil analyses

Forest floor N and P concentrations were determined by Technicon colorimetry (Pulse Instrumentation, Saskatoon, SK) following wet digestion of air-dried material. Percent organic matter of mineral soil samples was estimated by loss-on-ignition (6 h at 400°C). The pH was measured by H^+^ probe on aqueous suspensions (10:1 forest floor; 2:1 mineral soil) of air-dried soil subsamples. Fresh soil subsamples were extracted in 1 N KCl solution, filtered using Whatman No. 5 filter papers, and NH_4_^+^ + NO_3_^-^ concentrations (i.e., dissolved inorganic N (DIN)) were determined by Technicon colorimetry. Forest floor KCl extracts were further passed through 0.45 µm low protein-binding syringe filters prior to DIN analysis. Subsamples of these extracts were analyzed for total dissolved nitrogen (TDN), following persulphate oxidation to NO_3_^-^ [24]. Dissolved organic N (DON) was calculated as TDN minus DIN. Soil mineral N fertility (i.e. mineralizable N) in all samples was assessed from DIN concentrations following 30-day fresh soil incubations (20°C), after correcting for initial DIN concentrations [25].

Soil basal respiration (BR) was measured on fresh soil samples (ca. 10 g forest floor; 30 g mineral soil) sealed in 125 mL jars for 12 h. Headspace CO_2_ was then determined by Micro-GC (Chrompack, Middelberg, The Netherlands). Ambient CO_2_ and temperature were regularly recorded during assays. Ambient CO_2_ was subtracted from CO_2_ headspace concentrations, with differences adjusted to 22°C using Ideal Gas Laws and assuming Q_10_ = 2. The following day, the same soil samples were amended with glucose in order to estimate microbial biomass (MB) by substrate-induced respiration, using the protocol described by Bradley and Fyles [26]. Substrate-induced CO_2_ production was converted to MB using equations of Anderson and Domsch [27].

Condensed tannins in forest floor samples were measured colorimetrically using the proanthocyanidin assay (butanol-HCl hydrolysis), standardised against purified black spruce and *Kalmia* tannins for samples respectively collected under black spruce and *Kalmia* patches [28]. Briefly, samples were freeze-dried, ground in a mortar and pestle, and extracted twice with 70% aqueous acetone, which was then dried down under N_2_ for the determination of extractable tannins. The insoluble residue was dried under N_2_ for the analysis of residual tannins; butanol-HCl reagent was added directly to the residue. Total condensed tannin concentrations were calculated as the sum of extractable and residual tannins. Total phenolics were determined after rehydrating 0.5 mL aliquots of dried acetone-water extracts with 1.0 mL distilled water, then adding 0.5 mL Folin-Ciocalteu reagent (Sigma) and 2.5 mL of aqueous Na_2_CO_3_ (20% w/v). Solution absorbance (750 nm) was read on a spectrophotometer standardised against tannic acid (Sigma-Aldrich), as per Waterman and Mole [29].

The activities of β-glucosidase and acid phosphatase, two extracellular soil enzymes involved respectively in C and P cycling, were measured in forest floor extracts using a microplate-based assay described by Joanisse et al. [7]. Changes in fluorescence of 4-methylumbelliferone (MUB) that was cleaved by the enzymes from their respective substrates (4-MUB-β-d-glucoside, 4-MUB-phosphate) were measured at 10 min intervals over 60 min.

### Root and foliar analyses

Fine roots (<1-mm diameter) were collected from each uprooted *Kalmia* plant. These, along with subsamples of green and senescent *Kalmia* leaves and black spruce needles, were freeze-dried and milled to pass through a 40-mesh screen. An 80–100 mg aliquot of each ground subsample was encapsulated in Sn and analyzed for total C and N by high temperature combustion and thermoconductometric detection, using a Vario Macro dry combustion analyzer (Elementar Analysensyteme GmbH, Hanau, Germany). Freeze-dried and ground foliar subsamples were also hydrolyzed in butanol/HCl solutions and condensed tannins were quantified by the proanthocyanidin assay [30], using purified *Kalmia* tannins as standards [28]. Total phenolics were quantified by adding Folin-Ciocalteu reagent (Sigma-Aldrich, Oakville, ON) and aqueous 20% Na_2_CO_3_ to the rehydrated extracts, and comparing absorbance at 750 nm against those of tannic acid standards [29].

### Leaf litter decomposition

The remaining senescent *Kalmia* leaves were used to prepare 144 polyester litterbags (100 cm^2^; 1 mm mesh), each containing approximately 1.00 g of freeze-dried leaves. At the onset of the growing season (late-May), four litterbags were inserted into the surface (0–5 cm) forest floor layer of three black spruce and three *Kalmia* patches, at each of three spruce-lichen and three spruce-moss cutover sites. At each site, three litterbags per vegetation cover were retrieved after 3, 5, 12, and 25 months. At each of these dates, the litter in each bag was gently washed, freeze-dried and weighed to determine mass loss.

### Statistical analyses

For clarity, experimental factors are henceforth designated as (1) Site Type (i.e. spruce-moss cutovers, spruce-lichen cutovers or mature spruce-moss stands), (2) Vegetation (i.e. *Kalmia* or black spruce patches), (3) Horizon (forest floor, mineral Ae or podzolic B soil horizons) and (4) Time (i.e. time elapsed during decomposition assays).

NMR spectra of forest floor samples from spruce-moss and spruce-lichen cutovers were compared by two different means. First, we compared the distribution of relative areas between Site Types using PERMANOVA v.1.6 software [31]. This is a permutation-based program for analysing multivariate data on the basis of any distance measure [32,33]. We first permuted the data 499 times with Euclidean distance as our similarity measure. Due to our small sample sizes, the correct *P*-values (*P*_MC_) were obtained through Monte Carlo random draws from the asymptotic permutation distribution [34]. Following multivariate analysis, *t*-tests were done to compare each relative area of the selected regions between spruce-moss and spruce-lichen cutovers. As only one sample of mature spruce-moss forest floor was caracterized, no statistical comparisons were made with this sample and those from the cutovers.

Discriminant function analysis (DFA) was used to test whether Vegetation x Site Type groupings could be predicted based on a multivariate array of soil properties. DFA was performed separately for each soil horizon, after ensuring that all predictor variables were not highly correlated (i.e. |r| < 0.50). Thus, DFA for the forest floor was performed on all variables except residual tannins, which were highly correlated with extractable tannins. Eight variables were used for the mineral Ae horizon (moisture, % O.M., pH, DIN, mineralizable N, BR, MB and total phenolics) whereas six variables (% O.M., pH, DIN, mineralizable N, BR and MB) were used for the podzolic B horizon. Following DFA, one-way ANOVA and post-hoc Tukey tests were used to compare each classified group along the first two discriminant functions.

Linear mixed-effects models [35] were used to compare individual soil properties across the six Vegetation x Site Type combinations, using the identity of the 20 sites as a random variable. Separate models were run for the forest floor, Ae and B soil horizons. When Vegetation x Site Type interactions were significant, the effect of Vegetation was determined within each level of Site Type. In the absence of an interaction, the mixed model was rerun without the interaction term.

Fine root as well as green and senescent leaf chemistries were compared across Site Types using one-way ANOVAs followed by Tukey tests. A linear mixed-effects model (as described above) was applied to litter mass remaining in the decomposition experiment with Vegetation, Site Type and Time (and their interactions) as fixed factors, and site identity as a random factor. When interactions were present, *post-hoc* comparisons tested either simple-main effects (i.e. effect of Vegetation at each level of Site Type or Time, and vice versa), or simple-simple-main effects (i.e. effects of Vegetation at each level of Site Type and each level of Time).

For the decomposition study, percent mass remaining in the litterbags was analyzed using a linear mixed-effect model with Site Type, Vegetation and Time as fixed factors and the identity of each site (6 sites total) as a random factor.

Prior to all analyses, we verified that the data conformed to the assumptions of normality and homogeneity of variance; data were then ln-transformed when necessary to meet these assumptions. DFA, ANOVA and Tukey tests were all performed using SPSS 11.01 (SPSS Inc., Chicago, IL.) software. All linear mixed effects models were conducted in R statistical software [36]. Significance levels of all tests were set to *P <* 0.05, unless otherwise specified.

## Results

### Forest floor carbon chemistry

The relative intensities of the selected NMR spectral regions of forest floor samples are given in Table 1. Our interpretations of these spectral regions are based on findings from previous studies of litter and humus chemistry [22,23,37–40].

**Table 1:** Relative intensities (% total area) of ^13^C-CPMAS-NMR spectral regions of organic F-layers originating from three spruce-lichen and three spruce-moss cutovers, and from a single mature spruce-moss forest. **Values in parentheses are standard deviations of the mean**.

	alkyl-C	O-alkyl C		aromatic C		carbonyl-C	alkyl-C to O-alkyl-C ratio
		methoxyl and O-alkyl C	di-O-alkyl C	C-C and C-H	C-O and C-N	carboxyl, amide and ester C	
NMR chemical shift range (ppm)	(0–50)	(50–92)	(92–112)	(112–140)	(140–160)	(160–187)	(0–50)/(50–112)
Spruce-moss cutovers	24.5 (2.4)	41.8 (1.3)*	10.1 (0.9)	13.3 (0.7)^§^	4.4 (0.7)^§^	5.9 (0.8)*	0.47 (0.06)
Spruce-lichen cutovers	23.9 (1.7)	47.9 (2.9)*	11.2 (0.4)	10.1 (2.2)^§^	3.1 (0.8)^§^	3.9 (0.7)*	0.40 (0.02)
Mature spruce-moss forest	27.4	43.4	8.7	10.3	4.6	5.5	0.53

The symbols * and ^**§**^ **indicate differences between lichen and moss cutovers with P<0.05 and P<0.10 respectively.**

Given that a single bulk humus sample from mature spruce-moss forests was analyzed by NMR, it was not possible to including this Site Type in our statistical analyses. On the other hand, PERMANOVA revealed significant differences in the overall distribution of spectral regions when comparing forest floors from spruce-lichen and spruce-moss cutovers (F_1,4_ = 8.53, PMC = 0.038). More specifically, the peak in the alkyl region has two maxima at 30 and 33 ppm, characteristic of -CH_2_ in long chains, while the underlying broader intensity is due to a variety of -CH, -CH_2_ and -CH_3_ (i.e. methyl) structures. The peak at 33 ppm mainly represents long chain -CH_2_ from cutin, suberin and plant waxes, although microbial biomass may also contribute in this region. We found no significant differences in the relative intensity of this spectral region between spruce-lichen and spruce-moss cutovers. The largest peak in the O-alkyl region occurring at 73 ppm, as well as the sharp peak at 105 ppm, are mainly due to carbohydrates such as cellulose and hemicellulose. The relative intensity of this spectral region was significantly (P<0.05) higher in spruce-lichen than in spruce-moss forest floors. We found higher, albeit weakly insignificant (0.05 < P < 0.10), concentrations of aromatic C moieties (mainly at 130 ppm) in spruce-moss than in spruce-lichen forest floors. The largest peak in the carbonyl-C region occurred at 174 ppm and is ascribed to amide-C of proteins and the carboxyl groups of microbial and plant lipids. The relative intensity of this spectral region was significantly (P <0.05) higher in spruce-moss than in spruce-lichen forest floors. There were no significant differences in the alkyl-C to O-alkyl-C ratio.

### Predicting vegetation and site type based on aggregate soil properties

**Fig 1:**
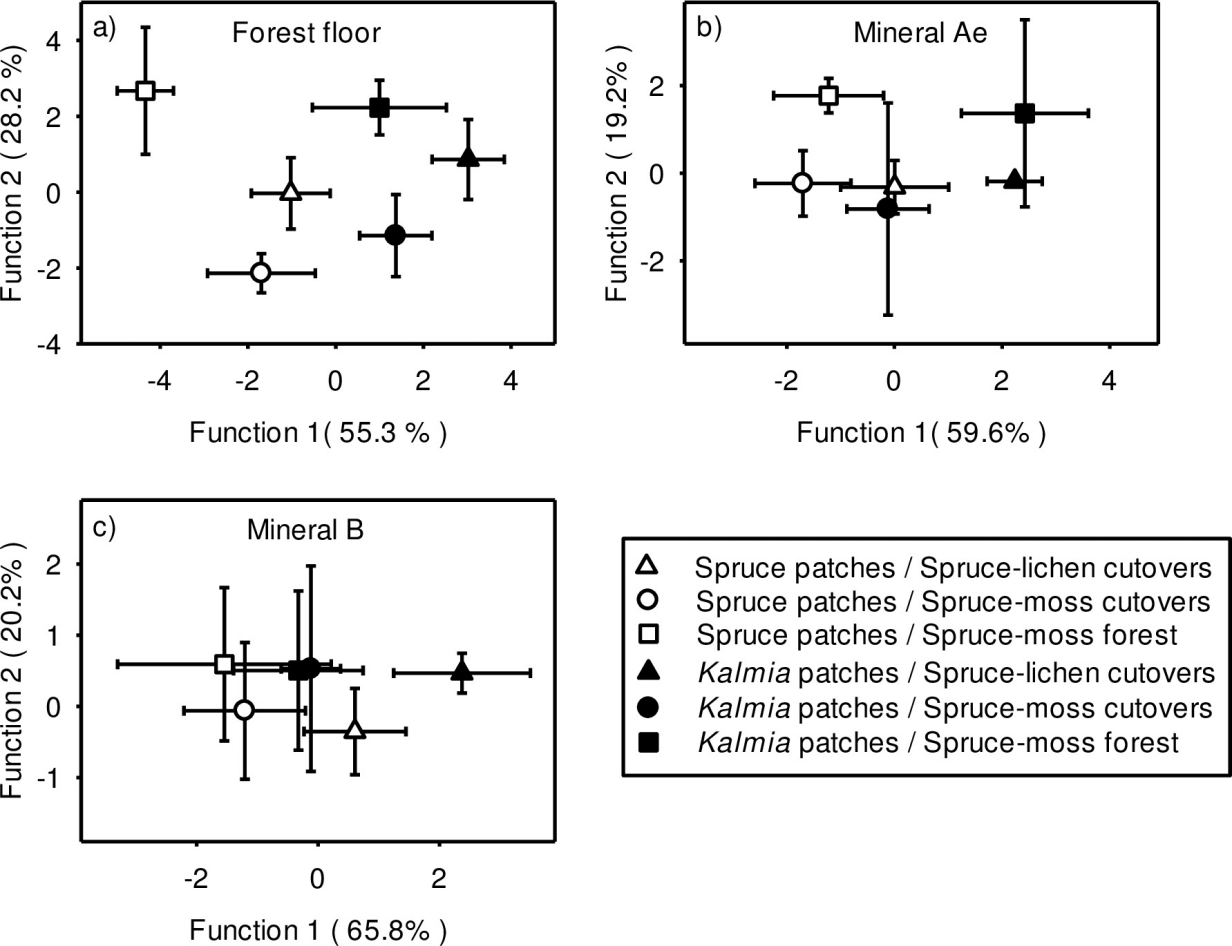
Ordination of the six vegetation x site type combinations along the first two discriminant functions (DFA), based on chemical and biochemical properties of (a) organic forest floor F-layer material, (b) Ae horizon mineral soil, and (c) B horizon mineral soils. Each point represents the mean of four or eight sites + 1 SD for each discriminant function.

For the organic forest floor F-layer, discriminant functions 1 and 2 explained 55% and 28% of the total variance in the data, respectively (Fig 1A). Overall, 95% of the samples were correctly classified according to Vegetation and Site Type. Function 1 mainly discriminated samples according to Vegetation, with four significant groupings: (1) spruce patches in spruce-moss forests, (2) spruce patches in spruce-lichen and spruce-moss cutovers, (3) *Kalmia* patches in spruce-moss forests and spruce-moss cutovers, and (4) *Kalmia* patches in spruce-lichen cutovers. Function 2 designated each Site Type as a significantly distinct grouping.

For the Ae horizon, discriminant Functions 1 and 2 explained 60% and 19% of the total variance, respectively (Fig 1B). Overall, 75% of the samples were correctly classified. Function 1 revealed three distinct groupings: (1) spruce patches in spruce-moss cutovers and in spruce-moss forests, (2) spruce patches in spruce lichen cutovers and *Kalmia* patches in spruce-moss cutovers, and (3) *Kalmia* patches in spruce-moss forests and spruce-lichen cutovers. Function 2 distinguished spruce patches in spruce-moss forests from spruce and *Kalmia* patches in each of the cutovers.

For the B horizon, discriminant Functions 1 and 2 explained 66% and 20% of the variance, respectively (Fig 1C). Overall, 62% of the samples were correctly classified. Function 1 revealed a significant difference between *Kalmia* patches in spruce-lichen cutovers and all other factorial combinations. Function 1 also revealed a significant difference between spruce patches in spruce-lichen cutovers and spruce patches in the other two Site Types. There were no significant groupings along Function 2.

### Effects of vegetation and site type on individual soil properties

#### Organic forest floor

**Fig 2:**
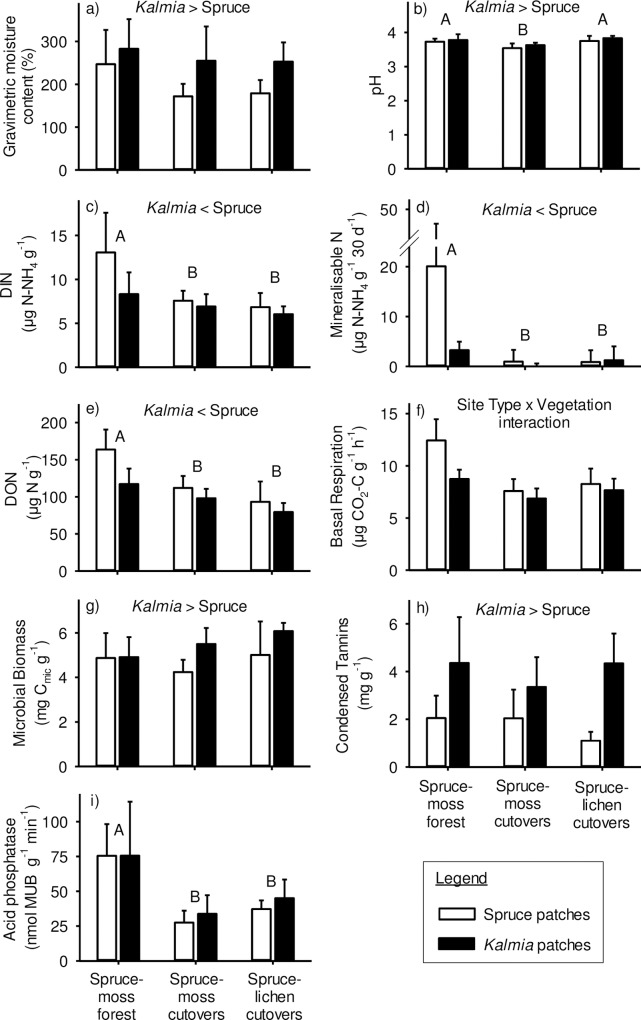
Forest floor F-layer properties that were significantly (P<0.05) affected by site type and/or vegetation. Different upper-case letters indicate significant differences between Site Types, whereas significant differences between spruce and *Kalmia* patches are written at the top of each frame. Significant Site Type x Vegetation interactions are explained in the Results section. Vertical lines = 1 SD.

Five forest floor properties were significantly affected by Site Type. More specifically, DIN, mineralizable N, DON and acid phosphatase activity were all higher in spruce moss forests than in both types of cutovers (Fig 2C, 2D, 2E and 2I). Forest floor pH, on the other hand, was significantly lower in spruce-moss cutovers than in the other two Site Types. Seven forest floor properties were significantly affected by Vegetation. More specifically, gravimetric moisture content, pH, MB and condensed tannins were all higher under *Kalmia* than under spruce patches (Fig 2A, 2B, 2G, and 2H). Conversely, DIN, mineralizable N and DON were higher under spruce than under *Kalmia* patches (Fig 2C, 2D and 2E). BR was significantly higher in spruce than in *Kalmia* patches, but only in mature spruce-moss forests (Fig 2F).

#### Mineral Ae and podzolic B horizons

**Fig 3:**
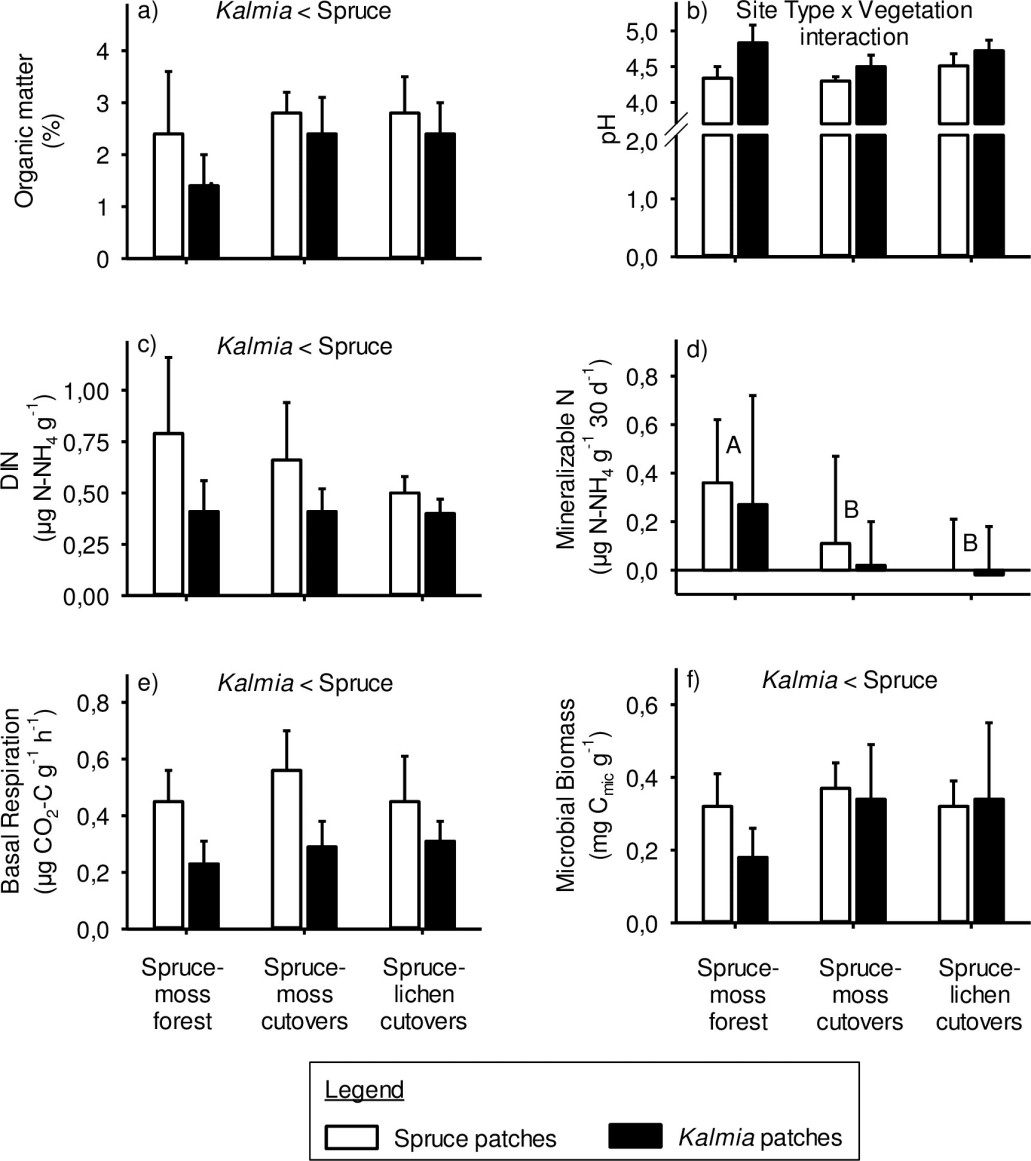
Mineral Ae horizon properties that were significantly (P<0.05) affected by site type and/or vegetation. Different upper-case letters indicate significant differences between Site Types, whereas significant differences between spruce and *Kalmia* patches are written at the top of each frame. Significant Site Type x Vegetation interactions are explained in the Results section. Vertical lines = 1 SD.

In the Ae horizon, % organic matter, DIN, BR and MB were all significantly higher in spruce than in *Kalmia* patches (Fig 3A, 3C, 3E and 3F). Mineralizable N, on the other hand, was higher in spruce-moss forests than in both types of cutovers (Fig 3D). Soil pH in spruce patches was significantly higher in lichen cutovers than in the other two Site Types (Fig 3B). By contrast, pH in *Kalmia* patches was significantly higher in spruce-moss forests than in spruce-moss cutovers (Fig 3B).

**Fig 4:**
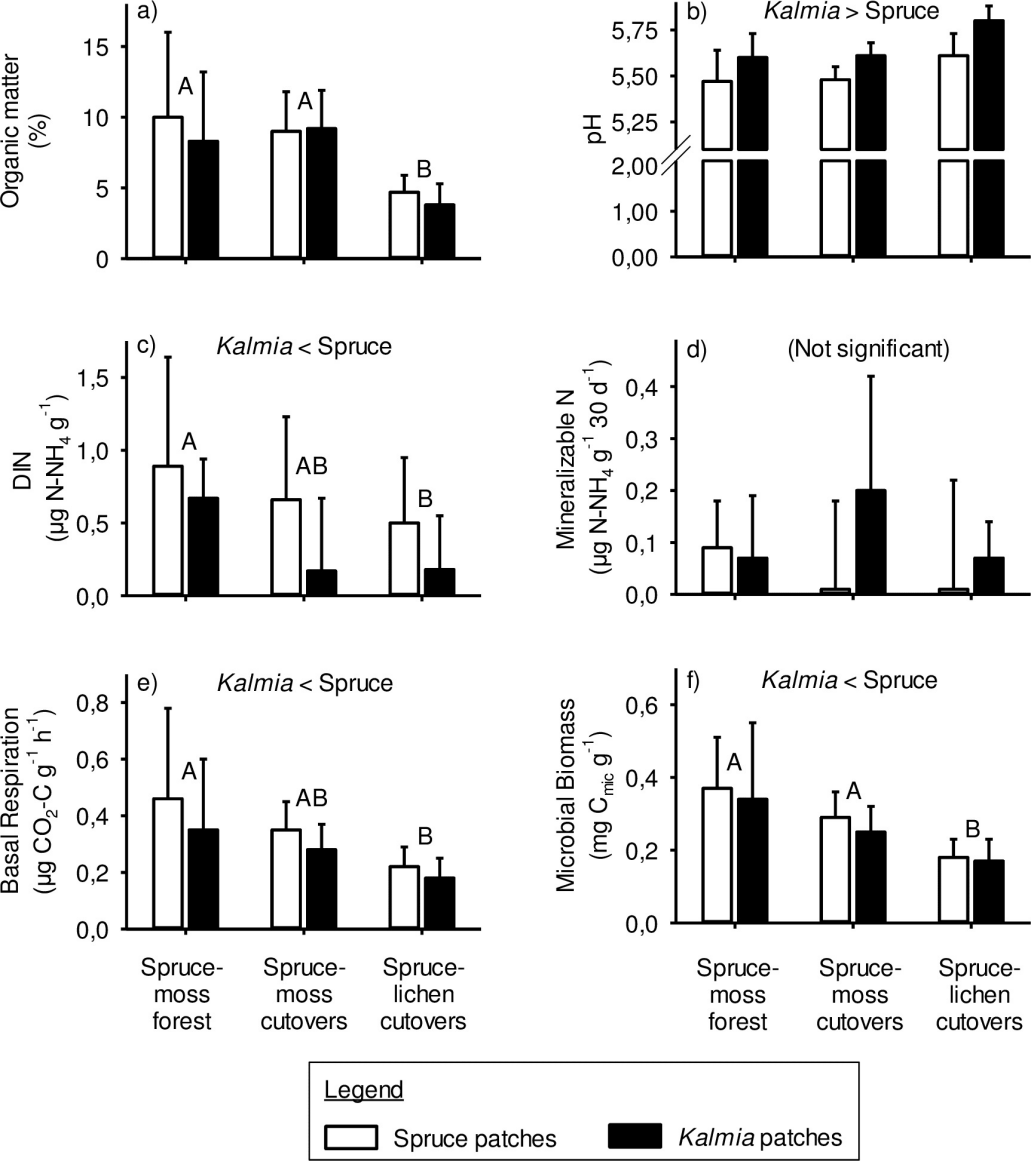
Mineral podzolic B horizon properties that were significantly (P<0.05) affected by Site type and/or vegetation. Different upper-case letters indicate significant differences between Site Types, whereas significant differences between spruce and *Kalmia* patches are written at the top of each frame. Vertical lines = 1 SD.

In the B horizon, soil pH was significantly higher in *Kalmia* than in spruce patches (Fig 4B). Percent organic matter and MB were significantly lower in spruce-lichen cutovers than in the other two Site Types (Fig 4A and 4F). DIN and BR were significantly higher in spruce-moss forests than in spruce-lichen cutovers (Fig 4C and 4E).

### Plant tissue chemistry

Both green and senescent *Kalmia* leaves had higher (P < 0.01) N, tannin and phenolic concentrations than green and senescent spruce needles respectively (Table 2). For *Kalmia* green leaves, total N was higher whereas total phenolics were lower in spruce-moss forests than in the two cutover types. The same pattern was observed in senescent *Kalmia* leaves for total N, although these effects were weakly insignificant (0.05 < P < 0.10). For spruce green needles, total phenolics were higher in spruce moss than in spruce-lichen cutovers.

**Table 2:** Chemical properties of fresh and senescent *Kalmia* leaves, *Kalmia* roots, and fresh and senescent spruce needles collected on different site types.

Vegetation	Site Type	Total N (mg g^-1^)	Total C (mg g^-1^)	Condensed tannins (mg g^-1^)	Total phenolics (mg g^-1^)
*Kalmia* green leaves	Spruce-lichen cutovers	10.2 (0.6) b	559.8 (5.3)	263.3 (12.7)	320.2 (5.7) a
	Spruce-moss cutovers	10.9 (0.8) b	555.2 (2.6)	272.5 (10.8)	332.1 (7.2) a
	Spruce-moss forests	13.2 (0.7) a	561.2 (3.4)	242.5 (28.3)	283.9 (25.0) b
*Kalmia* senescent leaves	Spruce-lichen cutovers	6.9 (0.9)	587.0 (6.6)	227.0 (25.9)	225.8 (21.9)
	Spruce-moss cutovers	6.5 (0.8)	573.6 (6.2)	222.7 (16.6)	216.7 (18.9)
	Spruce-moss forests	8.2 (0.5)	564.5 (0.2)	216.8 (19.9)	241.4 (12.3)
*Kalmia* roots	Spruce-lichen cutovers	5.2 (0.5)	554.0 (6.8)	234.6 (22.1)	111.8 (9.2)
	Spruce-moss cutovers	5.4 (0.2)	557.3 (5.4)	238.8 (19.6)	130.3 (13.4)
Spruce green needles	Spruce-lichen cutovers	7.2 (0.6)	529.5 (2.3)	93.9 (4.7)	100.7 (2.8) b
	Spruce-moss cutovers	7.0 (0.5)	536.3 (2.7)	103.2 (1.3)	107.9 (1.9) a
Spruce senescent needles	Spruce-lichen cutovers	2.3 (0.3)	580.0 (7.5)	48.5 (0.6)	96.0 (3.0)
	Spruce-moss cutovers	2.7 (0.4)	565.7 (2.0)	50.3 (1.9)	91.9 (1.9)

Values within a tissue type that are followed by different lower case letters differ significantly at P<0.05. Values represent means (n = 8 or 4) of each site type x tissue type combination, followed by 1 SD in parentheses.

### *Kalmia* litter decomposition

**Fig 5:**
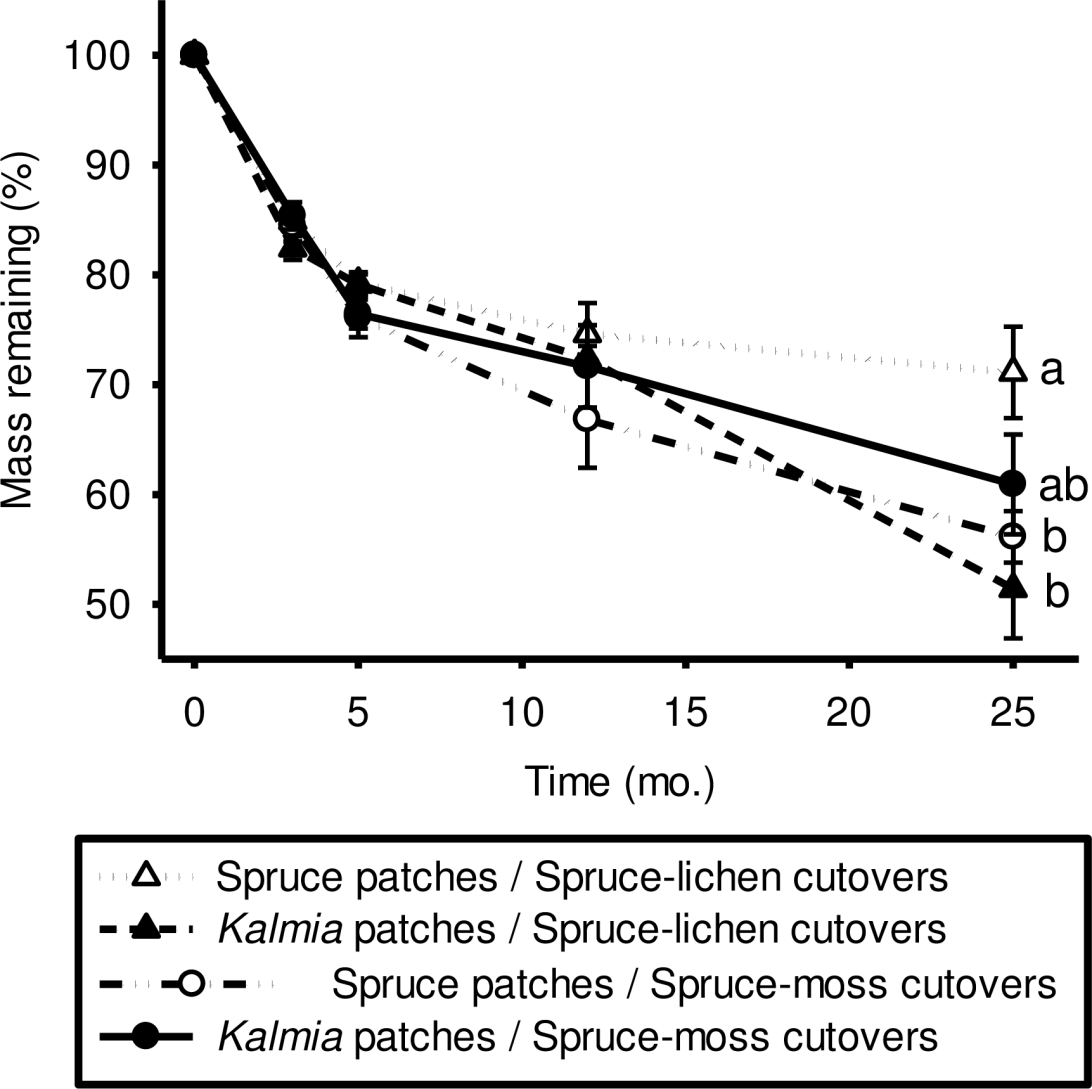
Percentage of *Kalmia* leaf litter mass remaining in litterbags during 25 months of *in situ* incubation; litter bags were either placed under *Kalmia* or under spruce patches, in both spruce-moss and spruce-lichen cutovers. Treatment assigned different lower case letters were significantly different after 25 months. Values represent the means of nine litter bags per sampling date ± 1 SD in parentheses.

Across all treatments, *Kalmia* leaf litter had lost 25% to 50% of initial mass following 25 months *in situ* incubation (Fig 5). Mass loss was significantly affected by Time, but also depended on Vegetation x Time and Site Type x Vegetation x Time interactions. After 25 months, more litter mass remained under spruce patches in spruce-lichen cutovers than under both spruce patches in spruce-moss cutovers and *Kalmia* patches in spruce-lichen cutovers.

## Discussion

### Forest floor carbon chemistry

We can only speculate on the factors contributing to different forest floor C chemistries in each Site Type, based on ^13^C-CPMAS-NMR spectra. The lower O-alkyl C (i.e. carbohydrates) and higher aromatic C peaks (i.e. lignin and other phenolics) in the spruce-moss cutovers are consistent with results from other studies [23,41] suggesting that these forest floors are more recalcitrant to decomposition than those in spruce-lichen cutovers. This is also consistent with results from Hagemann and Moronic [42], who performed a litterbag study in a boreal black spruce forest and showed higher decomposition rates for ground lichen than for feathermoss. It is also possible that spruce-moss forest floors accelerate the suberization of plant fine roots that proliferate in the forest floor, thus amplifying the aromatic signal of ^13^C-CPMAS-NMR spectra. For example, Sedia and Ehrenfeld [43] reported that oak seedlings growing in lichen patches had white fine roots whereas those in moss patches were of dark-brown colour. As for the carbonyl-C peaks in forest floor spectra, these are often ascribed to amide-C of proteins [22,23]. The fact that carbonyl-C peaks were higher in spruce-moss forest floors is consistent with higher concentrations of photosynthetic tissue and the presence of N fixing cyanobacteria in *Pleurozium* moss compared to fruticose lichens [44]. Regardless of the underlying reasons, the fact that distinct forest floor C chemistries were found in each Site Type portends to possible Site Type x Vegetation interactions in controlling soil properties and functions.

### Combining soil properties to discriminate site type and vegetation

Discriminant function analysis (DFA) confirmed that Site Type and Vegetation both exert a strong control on the chemical and biochemical profile of forest floors (Fig 1A). Function 1 correlated mainly with Vegetation whereas Function 2 correlated with Site Type. The segregation of the six Vegetation x Site Type combinations is less apparent with increasing soil depth (2B, and 2C). This is consistent with the fewer number of individual soil properties that are affected by Vegetation and Site Type with increasing soil depth (i.e. comparison of Figs 2, 3 and 4). Contrary to forest floor and surface Ae mineral horizons, individual properties of the mineral B horizon are mainly controlled by Site Type rather than by Vegetation (Fig 4). Given that spruce-moss and spruce-lichen ecosystems develop as a result of differences in drainage and geological parent material, it is logical that Site Type exerts a stronger influence on subsoil properties.

### Individual forest floor properties

The higher moisture content of forest floors beneath *Kalmia* may reflect a lower transpiration rate of *Kalmia* relative to black spruce, either as a result of lower photosynthesis, a higher water use efficiency, or both [6]. It is also possible that *Kalmia* slows the growth rate of plants in its immediate vicinity, thus lowering total evapotranspiration in its neighbourhood. Forest soils under *Kalmia* also had a higher pH than under spruce. However, the differences were small (ca. 0.1 pH units) and probably unimportant for soil nutrient processes.

Properties related to N cycling (i.e. DIN, mineralizable N and DON) and to microbial dynamics (i.e. BR and MB) were an order of magnitude higher in the forest floor F horizon than in mineral horizons. Given this high nutrient flux, black spruce trees and seedlings have evolved to produce most of their nutrient-absorbing fine roots in the forest floor [45,46]. However, the forest floor F layer is also where *Kalmia* fine roots are most abundant, sometimes comprising a biomass that is five times higher than regenerating black spruce seedlings [9]. Moreover, and as previously noted, *Kalmia* has a greater effect on forest floor properties than on the underlying mineral soil properties. The forest floor thus seems to constitute a “battleground” for nutrient acquisition between *Kalmia* and black spruce in forest understories and on cutovers. As we predicted, DIN and mineralizable N were lower beneath *Kalmia* than beneath black spruce, in line with the higher concentrations of condensed tannin that we found under *Kalmia*. This decrease in N cycling under *Kalmia* was most apparent, however, in mature spruce-moss forests than in the cutovers. The reason for this is not obvious, as soil condensed tannin concentrations under *Kalmia* were similar across Site Types. It may be due to the younger *Kalmia* clumps in the cutovers that have not yet reached their full potential for interfering with N cycling, as compared to long-established *Kalmia* clumps in mature forests that have accumulated tannin-protein complexes over a longer period.

The higher DIN and mineralizable N values in forest floors of mature forests, compared to both types of clearcuts, is consistent with studies reporting a loss of vigor by ericaceous shrubs after canopy closure [17]. It is also consistent with the lower total phenolic concentration of *Kalmia* green leaves in mature forests compared to clearcuts. Furthermore, higher DIN and mineralizable N values in mature forests mainly occurred under spruce patches, where microbial activity (BR) was higher, probably as a result of lower tannin and phenolic concentrations in spruce needles as compared to *Kalmia* leaves (Table 2). However, it is unclear why forest floor DON concentrations were also higher under spruce patches in mature forests than in cutovers. While DON concentrations have been shown to increase with forest succession, this phenomenon is usually attributed to increasing soil tannin inputs [16,47]. This is inconsistent with the lack of a Site-Type effect on soil tannins as well as the lower forest floor tannin concentrations found under spruce patches. Joanisse et al. [12] have challenged the interpretation of soil DON concentrations, however, based on the complex mixture of molecules that make up DON as well as their potentially transient nature.

In mature spruce-moss stands, forest floor BR was higher under black spruce than under *Kalmia*, but there was no effect of vegetation on MB. Conversely, BR in cutovers was unaffected by Vegetation whereas MB was higher under *Kalmia* than under spruce. Thus the CO_2_ output per unit microbial biomass, commonly referred to as the microbial metabolic quotient (*q*CO_2_), was consistently lower under *Kalmia* than under black spruce across all three Site Types. This higher C use efficiency under *Kalmia* may reflect microbial communities adapted to lower quality substrates [48].

Condensed tannins have been shown to reduce soil enzyme activity, a mechanism that may ultimately guide ecosystem structure and functions [7]. In our study, however, no such relationship was found between forest floor tannin concentrations and the activities of either acid phosphatase or B-glucosidase. The higher acid phosphatase activities that we found in mature spruce-moss forests compared to cutovers is consistent, however, with results from other studies [49]. Cline and Zak [50] ascribed this phenomenon to the accumulation of soil organic matter during secondary succession, leading to higher fungal β-diversity and extracellular enzyme activity.

### Individual mineral soil properties

The lower organic matter content of the mineral Ae horizon under *Kalmia* may be due to more recalcitrant forest floor humus under *Kalmia* that is less mobile (i.e. less leachable) than under spruce. This could also result from the higher pH under *Kalmia*, leading to higher flocculation and lower mobility of soil organic matter. A third possible reason may be that *Kalmia* produces less root biomass than black spruce in the mineral soil, as reported by Damman [51]. Thus the lower DIN, BR and MB values in the mineral Ae horizon under *Kalmia* may be due, to some extent, to lower organic matter content rather than lower organic matter quality.

While the effects of Vegetation on pH and DIN in the mineral B horizon were similar to those in the mineral Ae horizon, there remained some differences between these two horizons. The most notable of these are the lower organic matter, DIN, BR and MB values in the spruce-lichen cutovers compared to the other two Site Types. This possibly reflects lower C accumulation in these ecosystems, due to lower productivity and higher fire frequency [52].

### Plant tissue chemistry

There are three notable takeaways from the data presented in Table 2. First, there is little difference in plant tissue chemistry between spruce-moss and spruce-lichen cutovers. Although cutover type does have a statistically significant effect on total phenolic concentrations in spruce green needles, this 7% difference is quite small in comparison to the 300% difference in phenolic concentrations between spruce green needles and *Kalmia* green leaves. Thus the second takeaway from Table 2 is that tannin and phenolic concentrations are several times greater in green and senescent *Kalmia* foliage than in spruce needles. Although *Kalmia* leaves also contain higher concentrations of N than spruce needles, there is strong evidence that any potential increase in soil N cycling due to higher *Kalmia* litter N will be negated by the much higher tannin concentration of *Kalmia* litter [16]. The third takeaway from Table 2 is that *Kalmia* leaves have higher N and lower phenolic concentrations in mature forests than in cutovers. Although *Kalmia* may tolerate shade, canopy closure reduces condensed tannin concentrations of ericaceous shrubs [17,53,54]. This is likely due to lower photosynthesis resulting in proportionately more photosynthates being shunted for growth rather than for the production of secondary metabolites [55].

### *Kalmia* leaf litter decomposition

Generally, a greater availability of nutrients in the surrounding environment is expected to accelerate litter decomposition [56]. By contrast, *Kalmia* litter in our study decomposed more rapidly under the less fertile *Kalmia* patches than under spruce patches. A possible explanation for this is the phenomenon referred to as “home-field advantage” where microbial communities are locally adapted to decompose litter of the resident plant species [57,58]. It is notable that the difference in decomposition rates between *Kalmia* and spruce patches was greater in the xeric spruce-lichen cutovers than in the mesic spruce-moss cutovers. It is possible that the higher forest floor moisture in *Kalmia* patches amplified the home-field advantage in a xeric environment.

### Forest management implications

Although forest floor C chemistry in spruce-moss and spruce-lichen cutovers are chemically distinct, the small number of Site Type x Vegetation interactions that we observed suggests that managing ericaceous shrubs in both cutover types could be done in the same way. What is common to both ecosystems is that the spread of *Kalmia* contributes to the spatial heterogeneity of soil resources. Most notably is the fact that localized patches of *Kalmia* produce high concentrations of tannins and low cycling of mineral N. These effects are more concentrated in the forest floor, where black spruce seedlings obtain most of their nutrients. Furthermore, we have shown that the effect of *Kalmia* on forest floor properties increases as forest stands mature. Hence, the presence of *Kalmia* after CPRS could have long-term negative effects on forest productivity. Foresters should, therefore, seek ways to minimize the spread of *Kalmia* in black spruce understories before harvesting, or find ways to eradicate *Kalmia* on cutovers. One way to minimize *Kalmia* in the understory might be to eliminate the common practice of stand thinning in the early stages of stand development [59]. This opens the canopy and increases woody debris, possibly leading to the proliferation of ericaceous shrubs [4]. As for eradicating *Kalmia* on CPRS sites, the practice of mechanical scarification may be effective in giving a competitive lead to regenerating black spruce seedlings over *Kalmia* [8].

## Supporting information

S1 Data Files (XLSX)
